# A 72-year-old woman with an uncorrected tetralogy of Fallot presenting with possible pulmonary endocarditis: a case report

**DOI:** 10.1186/1752-1947-7-150

**Published:** 2013-06-02

**Authors:** Pedro Sousa, Walter Santos, Nuno Marques, Pedro Cordeiro, Rui Ferrinha, Salomé Pereira, Ana Almeida, Ilídio Jesus

**Affiliations:** 1 Cardiology Department, Faro Hospital, Rua Leão Penedo, Faro 8000, Portugal; 2 Faculty of Medicine of Lisbon University, SPRM, Lisbon, Portugal

**Keywords:** Infective endocarditis, Longevity, Pulmonary valve, Tetralogy of Fallot, Uncorrected

## Abstract

**Introduction:**

Tetralogy of Fallot is one of the most common forms of cyanotic congenital heart disease and in the absence of surgical correction it has an elevated early mortality, with most patients dying in childhood.

The authors reported this case because of the unusual course of an uncorrected tetralogy of Fallot. There are only a few reports of patients with an uncorrected tetralogy of Fallot who reach an advanced age and to the best of our knowledge this is the first case report of a possible endocarditis in a patient with an uncorrected tetralogy of Fallot who is older than 70 years.

**Case presentation:**

The authors present a case of a 72-year-old Caucasian woman with uncorrected tetralogy of Fallot who was admitted with fever and heart failure to our Cardiology Department with possible infective endocarditis.

**Conclusions:**

The longevity of this patient is probably due to the association between a large ventricular septal defect, a balanced subpulmonary stenosis and to the presence of systemic hypertension. After empiric antibiotic therapy, the patient was discharged and no surgical intervention was performed due to her previous benign evolution.

## Introduction

Tetralogy of Fallot (TOF) was first described in 1888 by Étienne-Louis Arthur Fallot and is characterized by the presence of four anatomical anomalies: ventricular septal defect (VSD), pulmonary stenosis/outflow obstruction of the right ventricle (RV), RV hypertrophy and an aorta that overrides the VSD. The primary lesion is the head and anterior deviation of the infundibular septum, which ultimately leads to the four classical alterations [1,2].

TOF is one of the most common forms of cyanotic congenital heart disease [3]. Prolonged survival without surgical intervention is rare. Epidemiological studies and autopsy records demonstrated that only 2% of all patients with TOF reach the fourth decade of life [4] and survival beyond the seventh decade of life is even rarer [5]. Prolonged survival of patients with uncorrected TOF is often associated with a well-developed left ventricle [6], initially mild pulmonary stenosis that progresses or adaptations that ameliorate the right-to-left shunting, such as systemic to pulmonary collaterals, persistent patent ductus arteriosus or systemic hypertension [1]. The main causes of death are chronic congestive heart failure, secondary to the long-standing pressure overload and consequent pathological changes in the RV, and arrhythmias [6].

TOF patients carry a high risk for the development of infective endocarditis (IE) [7], which is a serious and fatal complication in adults with congenital heart disease [8,9]. The incidence of IE in patients with TOF submitted to surgery, either corrective or palliative, is high (around 18%), whereas in patients with uncorrected TOF this incidence is small (around 4%) [10,11]. The most common organisms are streptococci, followed closely by staphylococci [8,9].

In the current era, late surgical repair of TOF is recommended because the surgical risk is considered to be acceptable and the long-term results have been gratifying in the rare series reported: short- and long-term mortality rates of 3 to 16% and 6 to 24%, respectively [12-14].

The authors describe a case of a 72-year-old woman with an uncorrected TOF with possible IE.

## Case presentation

The authors report a case of a 72-year-old Caucasian woman with a history of controlled hypertension, followed in the cardiology consultation for uncorrected TOF. In the past, she had a pregnancy that elapsed without complications and gave birth to a healthy daughter. Currently, she usually complains of dyspnea, and is in class II of the New York Heart Association (NYHA).

**Figure 1:**
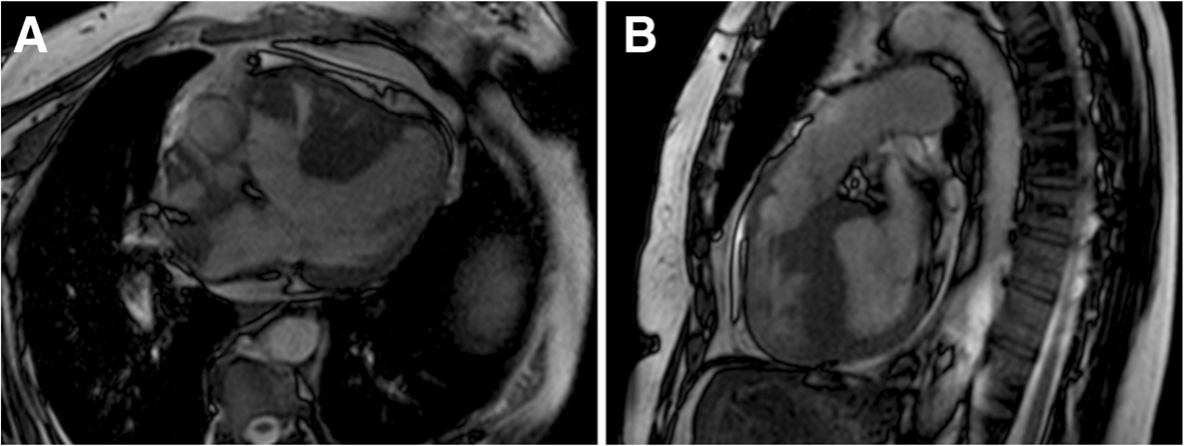
**Cardiac magnetic resonance imaging confirming the diagnosis of tetralogy of Fallot. A:** Ventricular septal defect and overriding aorta over the septum. **B:** Right ventricular hypertrophy and subpulmonary stenosis.

During follow up in the cardiology consultation, she underwent a cardiac magnetic resonance imaging (Figure 1), which revealed a large subaortic VSD, with a diameter of 26mm and bidirectional flow (QP:QS = 0.9); an overriding of the aorta over the septum ≤50%; a marked hypertrophy of the RV with subpulmonary stenosis (maximum gradient of 31mmHg) and moderate pulmonary regurgitation; a dilatation of the pulmonary artery and its branches; and a non-dilated left ventricle with preserved systolic function. It also revealed the absence of patent ductus arteriosus.

**Figure 2:**
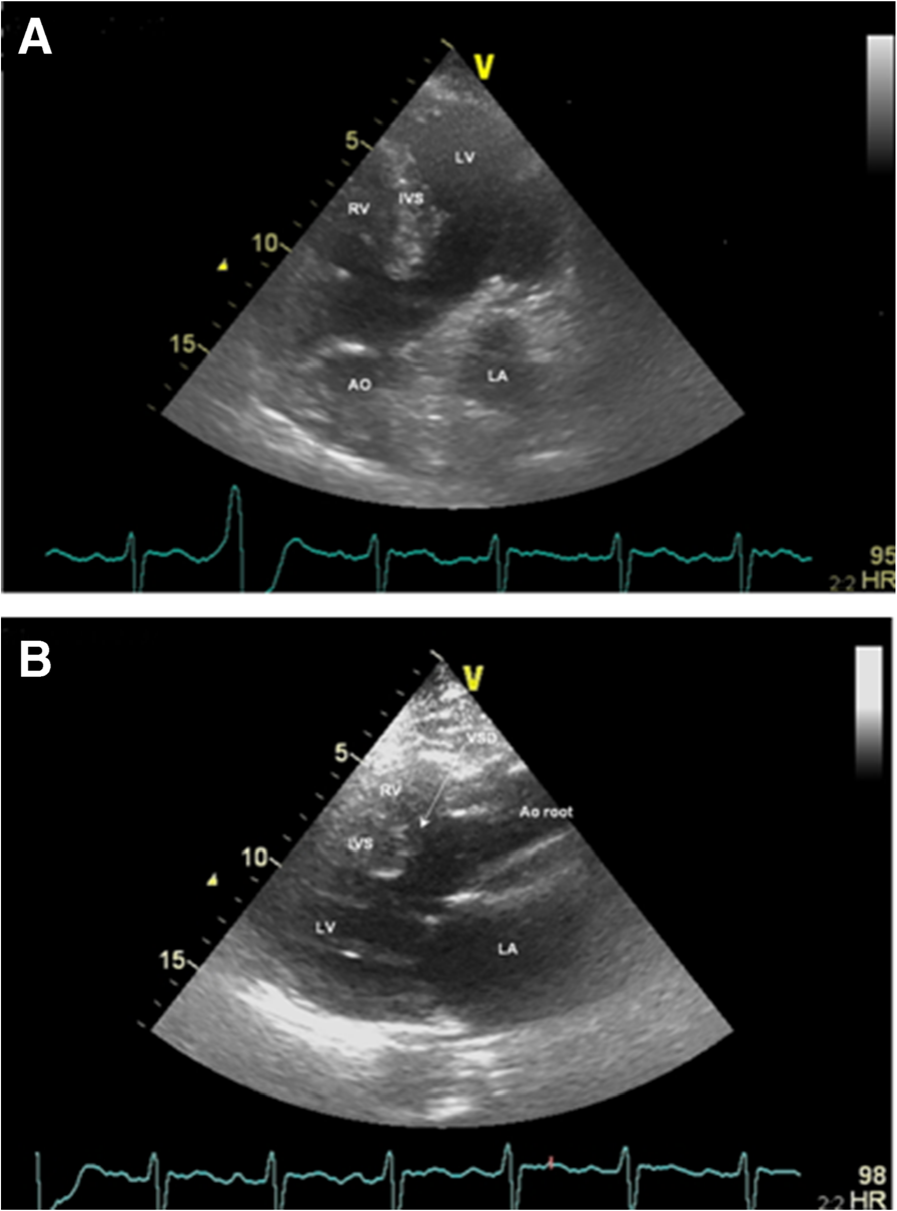
**Transthoracic echocardiogram revealing some of the characteristics of tetralogy of Fallot. A:** Five chamber view with the presence of the ventricular septal defect (VSD) and the overriding aorta over the septum. **B:** Parasternal long axis view revealing the VSD, the overriding of the aorta over the septum and also the right ventricle (RV) hypertrophy. AO, aorta; IVS, interventricular septum; LA, left atrium; LV, left ventricle.

**Figure 3:**
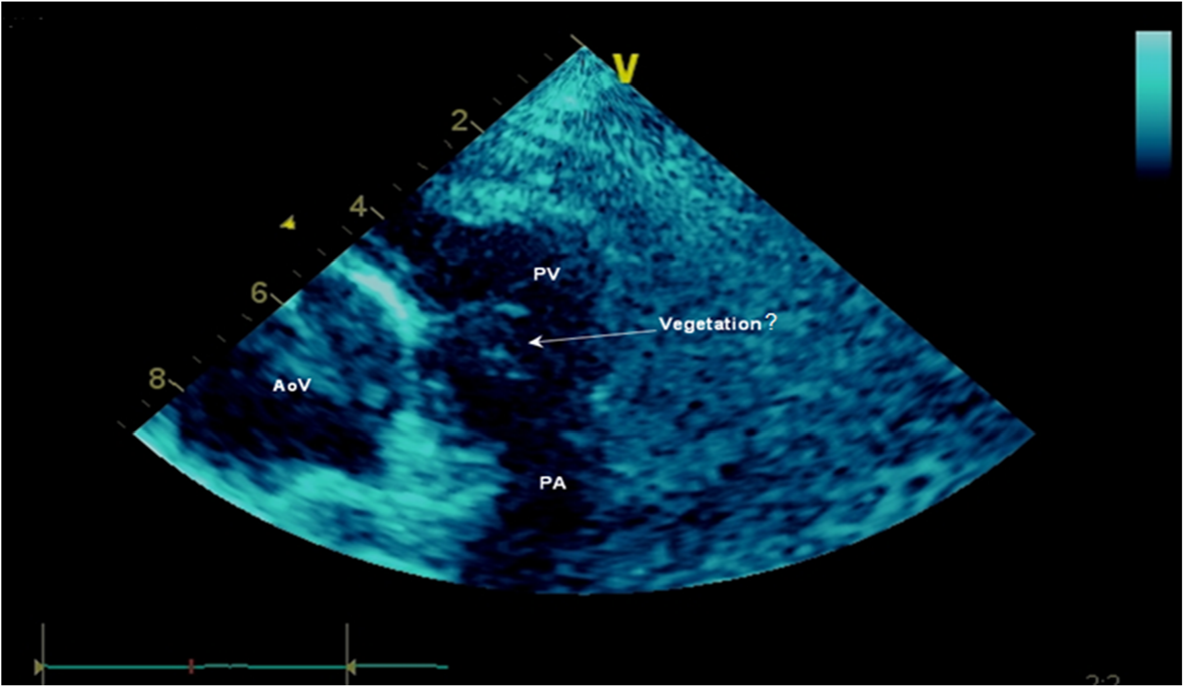
**Transthoracic echocardiogram revealing an erratic structure in the pulmonary valve suggestive of vegetation.** AoV, aortic valve; PA, pulmonary artery; PV, pulmonary valve.

Approximately 1 year prior to her admission to hospital, she had started treatment of dental caries and a tooth extraction was carried out under antibiotic prophylaxis. Five months prior to admission, she reported persistent predominantly vespertine low-grade fever episodes associated with asthenia and weight loss. The patient sought medical assistance several times and was medicated with four antibiotics for the proposed diagnosis of respiratory and urinary infections. Given the persistence of fever and progressive worsening of heart failure, she was brought to a hospital. At admission, she was in class III of the NYHA and presented with fever, slight peripheral cyanosis, finger clubbing despite oxygen saturation of 91%, a regular pulse of 95 beats per minute and a blood pressure of 149/70mmHg. Cardiac examination revealed a single second heart sound, a systolic thrill and loud systolic ejection murmur (grade IV) at the base of her heart. An electrocardiogram showed normal sinus rhythm with first degree atrioventricular block and incomplete right bundle branch block. Blood picture showed leukocytosis (white blood cells: 17,700), elevation of C-reactive protein with high sedimentation rate and rheumatoid factor (RF) and mild anemia (hemoglobin 11.7g/L and hematocrit 0.35L/L). Transthoracic echocardiography (TTE) showed the components of TOF (Figure 2) and an echodense, irregular and mobile mass, 10mm long and 3mm wide, adherent and downstream to the pulmonary valve suggestive of vegetation (Figure 3) without associated regurgitation. Therefore, she was hospitalized in our Cardiology Department for suspected IE.

Blood cultures were collected and empiric antibiotic therapy with vancomycin, gentamicin and ciprofloxacin was started. No vascular or immunological phenomena (except RF) were detected. All blood cultures collected, at admission and during antibiotic therapy, were negative inclusively for atypical organisms. Serology tests were also negative. Computed tomography images revealed no signs of cerebral, thoracic or abdominal embolizations.

Transesophageal echocardiography (TEE) was not able to confirm or exclude the presence of pulmonary vegetation given the complex heart anatomy. Despite this, because the patient had a predisposing heart condition, fever and elevated RF, it was decided to maintain the empiric antibiotic therapy.

**Figure 4:**
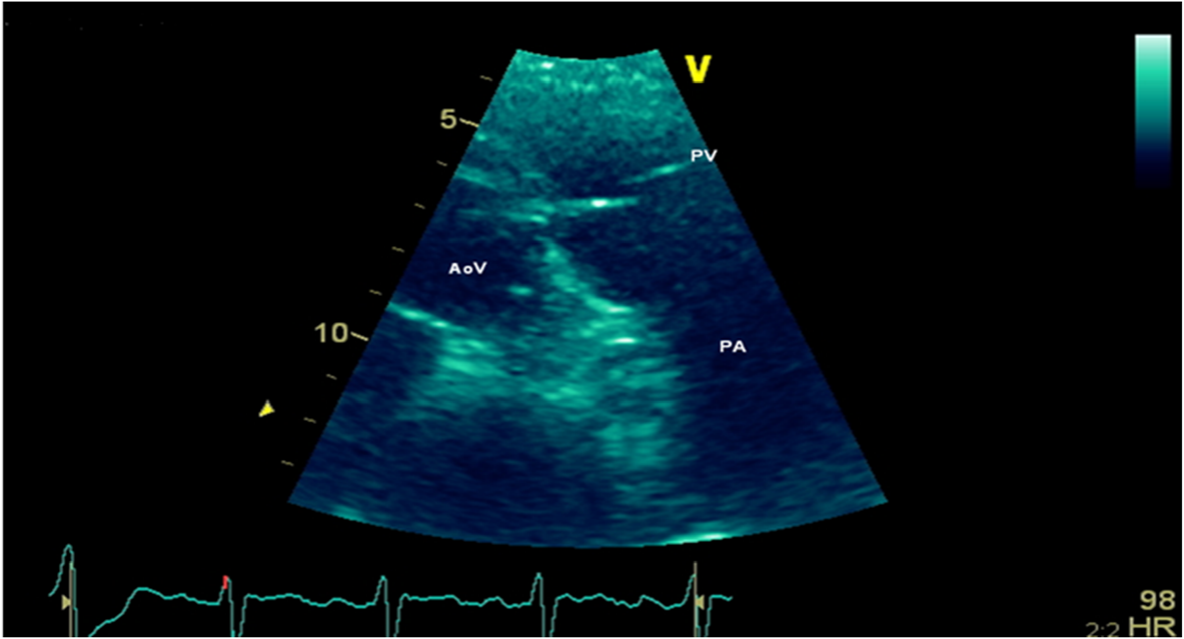
**Transthoracic echocardiogram showing the pulmonary valve at discharge, after 4 weeks of empiric antibiotherapy.** AoV, aortic valve; PA, pulmonary artery; PV, pulmonary valve.

After 4 weeks of antibiotic therapy, the fever and the elevation of the inflammation markers had resolved, the patient was back in class II of the NYHA and no images suggestive of vegetation were detected on TTE (Figure 4). Therefore, the patient was discharged.

One year after her hospitalization she remained apyretic and without any images suggestive of vegetation on TTE.

## Discussion

The longevity of this patient is possibly explained by the unique combination of a large VSD, a well-developed and normally functioning left ventricle and a balanced subpulmonary stenosis, mild enough not to cause severe hypoxia, but important enough to counterbalance the left ventricle and to protect the pulmonary vasculature [3]. Systemic hypertension may also play a role in the balancing of pressure between the two ventricles [1].

The diagnosis of IE was considered possible because, although the image of the vegetation on TTE was downstream to the pulmonary valve and not confirmed by TEE, three minor modified Duke criteria were present: predisposing heart condition, body temperature above 38°C and elevated RF [15]. The favorable outcome of the patient when treated with empiric antibiotic therapy for IE also supports this diagnosis. Predisposal procedures, like treatment of dental caries and tooth extraction, even under antibiotic prophylaxis, may have led to bacteremia [15]. Blood cultures were negative probably due to the previous antibiotic therapy.

Finally, considering the benign course of this congenital heart disease, that allowed the patient to lead an autonomous life up to 72 years, and because surgery for uncorrected TOF in elderly patients is associated with increased mortality, we opted for conservative medical treatment and no invasive diagnostic or therapeutic measures were undertaken for TOF.

## Conclusions

This case report highlights the unusual course of an uncorrected TOF in a 72-year-old woman and also emphasizes the importance of TTE in the diagnosis of TOF and particularly of IE in these patients. In this case, TTE allowed the detection of vegetation in the pulmonary valve, despite the complex heart anatomy of the patient, and it was crucial to the diagnosis of IE and subsequent noninvasive management and favorable outcome of this patient.

## Consent

Written informed consent was obtained from the patient for publication of this case report and accompanying images. A copy of the written consent is available for review by the Editor-in-Chief of this journal.

## Competing interests

The authors declare that they have no competing interests.

## Authors’ contributions

AA performed and analyzed the cardiac magnetic resonance. PC and RF evaluated the echocardiographic images. WS, NM, SP and IJ were major contributors in writing the manuscript. PS wrote the manuscript. All authors read and approved the final manuscript.
